# The Role of Lifestyle Intervention in Female Fertility: A Modifiable Factor for Preconception Health

**DOI:** 10.3390/nu17132101

**Published:** 2025-06-25

**Authors:** Marisa Donato, Antonio Capalbo, Elisena Morizio, Rosa Maria Fratini, Lucrezia Pilenzi, Francesco D’Antonio, Liborio Stuppia, Ester Vitacolonna, Valentina Gatta, Fani Konstantinidou

**Affiliations:** 1 Department of Neuroscience, Imaging and Clinical Sciences, School of Medicine and Health Sciences, “G. d’Annunzio” University of Chieti-Pescara, 66100 Chieti, Italy; mdonato1@unite.it (M.D.); antonio.capalbo@unich.it (A.C.); rmfratini@unite.it (R.M.F.); lucrezia.pilenzi@studenti.unich.it (L.P.); liborio.stuppia@unich.it (L.S.); v.gatta@unich.it (V.G.); 2 Unit of Molecular Genetics, Center for Advanced Studies and Technology (CAST), “G. d’Annunzio” University of Chieti-Pescara, 66100 Chieti, Italy; elisena.morizio@unich.it; 3 Department of Bioscience and Technology for Food, Agriculture and Environment, University of Teramo, 64100 Teramo, Italy; 4 Reproductive Genetics, Juno Genetics Italy, 00197 Rome, Italy; 5 Department of Sciences, “G. d’Annunzio” University of Chieti-Pescara, 66100 Chieti, Italy; 6 Center for Fetal Care and High-Risk Pregnancy, Department of Obstetrics and Gynaecology, “G. d’Annunzio” University of Chieti-Pescara, 66100 Chieti, Italy; francesco.dantonio@unich.it; 7 Department of Medicine and Aging, School of Medicine and Health Sciences, “G. d’Annunzio” University of Chieti-Pescara, 66100 Chieti, Italy

**Keywords:** nutrition, lifestyle, physical activity, preconception health, infertility, IVF, PUFAs, gene expression

## Abstract

Infertility is a growing global phenomenon affecting millions of individuals and is characterized by multifactorial causes, including both lifestyle and environmental factors. These include smoking, chronic exposure to environmental pollutants, stress, excessive caffeine or alcohol intake, drug use, improper eating habits and physical inactivity. The potential to modify these behaviors has gained increasing interest due to its impact on reproductive health and its role in mitigating infertility. Preconception counseling has also emerged as a fundamental strategy, providing education and risk assessments to improve pregnancy outcomes. Among lifestyle factors, nutrition, body composition and physical activity significantly influence female fertility, emphasizing the strong connection between metabolism and reproductive function. Supplementation with anti-inflammatory nutrients, such as omega-3 polyunsaturated fatty acids (*n*-3 PUFAs), a key component of the Mediterranean diet, may offer benefits for female fertility, partially through the modulation of gene expression in reproductive tissues. However, the specific mechanisms linking diet and fertility remain unclear. The primary objective of this review is to explore how the modification of selected lifestyle factors, with particular reference to dietary habits, may positively influence the female reproductive system and improve fertility and pregnancy-related outcomes.

## 1. Introduction

Infertility is a complex pathological condition that affects millions of people of childbearing age worldwide, especially in industrialized regions [1]. It is defined by the failure to get pregnant after 12 months or more of unprotected sexual intercourse and constitutes a significant psychological, physical and economic burden for people trying to conceive [2].

Fertility potential can be influenced by several modifiable lifestyle factors, including cigarette smoking [3,4], chronic exposure to environmental pollutants, stress, high caffeine or alcohol intake, drug use and poor dietary habits. These factors play a pivotal role in the development of infertility and have generated growing interest, particularly due to the opportunity to improve both general and reproductive health through behavior modification [5].

An important tool that should be taken into consideration is preconception counseling aimed at health education and promotion for individuals and couples, with the potential to improve pregnancy-related outcomes [6]. It allows for the identification and modification of health risks to the potential mother prior to conception, as well as education on approaches that could reduce these risks, such as diet, nutrition and lifestyle. Therefore, preconception counseling has the potential to notably reduce maternal and newborn morbidity and mortality [7].

Body composition, physical activity (PA) and nutritional habits are key elements that might impair female fertility, which are due to the close relationship and reciprocal regulation between female reproduction and metabolism [8,9,10]. Given the increasing number of women dealing with fertility problems, often in combination with overweight and obesity, understanding how PA could help with reproductive health problems, alone or in combination with other lifestyle interventions, is of vital importance. Physical training is known to offer considerable health benefits, contributing to a better quality of life and, potentially, improved fertility outcomes [11,12,13].

Likewise, nutrition plays a crucial role in maintaining systemic and reproductive health. Unbalanced diets, particularly those that are hypercaloric, can increase inflammation, disrupt hormonal balance and lead to oxidative stress, all of which can impair fertility [14,15,16]. Certain dietary patterns, such as the Mediterranean diet and “pro-fertility” diets, have been associated with better reproductive outcomes, largely due to their anti-inflammatory and antioxidant properties [17,18,19].

Supplementation with omega-3 polyunsaturated fatty acids (*n*-3 PUFAs), such as eicosapentaenoic acid (EPA) and docosahexaenoic acid (DHA), may improve fertility by modulating prostaglandin synthesis, steroidogenesis and gene expression in reproductive tissues [20,21,22]. Despite promising evidence, the exact biological mechanisms underlying the link between diet and fertility remain unclear and warrant further research [23].

The high prevalence of infertility, along with the substantial financial burden and limited access to treatment, highlights the importance of broadening our understanding of how lifestyle interventions can support female reproductive health.

In this narrative literature review, a computer-assisted search was conducted using the PubMed and Google Scholar databases to identify relevant publications related to the topic of interest. The following selection criteria were applied: original articles and narrative, systematic or meta-analytic reviews written in English, as well as the use of keywords such as “nutrition”, “lifestyle”, “female infertility”, “preconception health”, “physical activity”, “gene expression” and “PUFAs”, alone or in combination with each other. The initial query was also limited to articles published from 2014 to 2024, which specifically examined the role of lifestyle intervention, with a particular reference to nutrition and physical activity, on the improvement of female fertility at a preconception stage. After examining all the articles, a final selection was made based on those that were aligned with the themes and subthemes of the narrative review. Finally, their reference lists were also evaluated in order to detect other potentially related studies or information that could be further included in this review.

**Figure 1:**
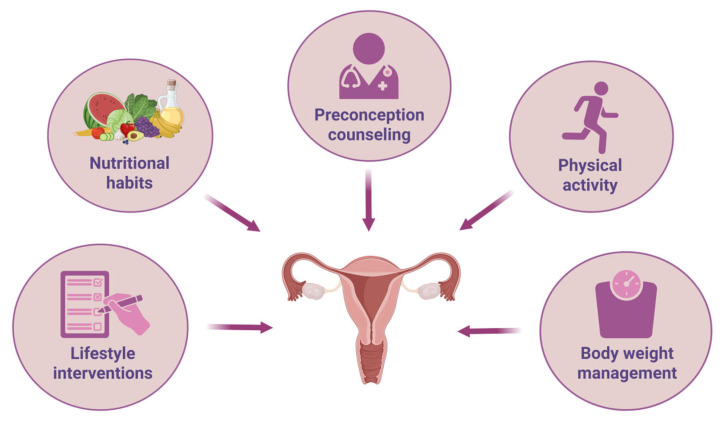
Types of preconception intervention that can improve reproductive fitness.

The main objective of this review is to explore how modifying specific lifestyle factors, like dietary choices and PA, can positively influence the female reproductive system and improve fertility and pregnancy-related outcomes. Moreover, it aims to highlight how certain nutrients and lifestyle interventions may impact gene expression in key reproductive tissues, thus contributing to the regulation of reproductive function (Figure 1).

## 2. Body Weight Management and Improvement of Fertility: Lifestyle Interventions

Obesity and overweight in females have a detrimental effect on reproduction, including ovulatory and menstrual function, fecundity and fertility rates, infertility treatment success rates and obstetric outcomes [24]. Excess weight represents a modifiable risk factor that could affect the reproductive potential of women that want to conceive. It has been demonstrated that lifestyle modification, through diet intervention and physical training, for weight management prior to pregnancy, may have the potential to provide the opportunity to improve fertility rates and provide a healthy pregnancy to the patient.

Beginning gestation at an ideal weight reduces the risk of hypertensive disorders, diabetes, thromboembolic diseases, preterm labor and cesarean section. In this context, preconception counseling constitutes an important tool in order to assist women to conceive in the healthiest condition possible, as well as an opportunity to educate the patient on their weight status and its link to reproductive health. It has been supported that women should be encouraged to reduce their caloric intake, achieve a body mass index (BMI) in the normal range before pregnancy and receive counseling on the components of a fertility-related healthy diet [25].

Lifestyle changes comprise a series of interventions that involve behavioral modifications, caloric restriction, increased PA and, occasionally, medical treatments. Weight management may be achieved by changing eating habits, creating a negative energy balance between hypercaloric diets and increased physical activity [24], which has been shown to potentially improve the success rates of conception or live birth through short- or long-term interventions. Combined types of interventions are not exclusively beneficial prior to a specialized fertility treatment but are also designed to improve overall health and promote spontaneous conception [26].

Conventional lifestyle interventions result in about a 3–7% weight reduction after 4–6 months, even though over 80% of the patients experience weight recovery after 1 year [27]. The guidelines for exercise advise women attempting to conceive to aim to gradually increase their PA to an average of 10,000 steps per day and/or engage in moderate aerobic exercise for 150 min per week [24].

It has also been demonstrated in women affected by polycystic ovarian syndrome (PCOS) that cognitive behavioral therapy lifestyle interventions can increase the number of women that achieve a healthier body weight [28]. Combined with an additional dietary intervention, this may represent a key element in the process of weight management to improve fertility outcomes. Even though it has been suggested that body weight interventions should be considered for infertile obese and overweight women, currently, there are very few validated trials that support these approaches and recommendations for an optimal lifestyle able to improve fertility.

### 2.1. Diet and Improvement of Fertility

#### 2.1.1. Pre-Treatment Dietary Patterns and Assisted Reproductive Technology (ART) Outcomes

Diet and nutritional habits are correlated with female fertility, and, in particular, specific food groups and dietary patterns have been shown to have a significant effect on reproductive health. Nevertheless, despite growing interest in this area, few studies have examined the role of dietary patterns in the outcomes of ART, and there are still no available specific dietary guidelines.

In order to evaluate the relationship between dietary patterns and the outcomes of ART, Gaskins et al. [29] examined 357 women enrolled in the prospective Environment and Reproductive Health (EARTH) study, who underwent 608 ART cycles. A validated food frequency questionnaire was employed and completed before the treatment to evaluate the adherence to the Mediterranean Diet (MedDiet), to the alternate Healthy Eating Index 2010 (aHEI2010), to the Fertility Diet (FD) and to a “pro-fertility” diet. The “pro-fertility” diet was characterized by higher intake of supplemental vitamin B12, vitamin D, folic acid, low-pesticide fruits and vegetables, dairy products, soy-based foods, whole grains and seafood. The results showed that adherence to this dietary pattern was associated with an increased probability of live birth following ART due to the fewer cycles that failed prior to embryo transfer and reduced pregnancy loss. On the other hand, MedDiet was linked to improved live birth outcomes without any additional benefits beyond that. The aHEI-2010 and the Fertility Diet were not consistently associated with ART success.

In a study conducted on 244 non-obese women (22–41 years of age; BMI < 30 kg/m^2^) who underwent their first in vitro fertilization (IVF) treatment in an assisted conception unit in Athens, Greece, the influence of habitual dietary intake and lifestyle on fertility outcomes was assessed [30]. Diet, particularly their adherence to the MedDiet, was evaluated before the IVF treatment through the validated Mediterranean diet score (MedDietScore). Karayiannis et al. found that their adherence to this healthy diet during the 6-month period before IVF was related to an improved likelihood of clinical pregnancy and live birth rate. Particularly, the beneficial effect of MedDiet was visible among women younger than 35 years involved in the study. Hence, these results show that observing a Mediterranean-type diet can potentially improve the chance of a successful pregnancy and delivering a live baby for women undergoing IVF treatment.

The relationship between pre-treatment dietary patterns and ART outcomes was also evaluated in subfertile couples (*n* = 161), who had been given a questionnaire based on their lifestyle and nutrition habits before IVF/intracytoplasmic sperm injection (ICSI) treatment [31]. The study identified two main dietary patterns among the patients involved. The first group, whose diet was labeled as “health conscious–low processed”, had a high intake of vegetables, fruits, legumes, whole grains and fish, but a low consumption of meat products, snacks and mayonnaise. The second group, whose diet was denominated as “Mediterranean”, was characterized by a high intake of vegetables, legumes, fish and vegetable oil, but a low intake of processed foods. Couples adhering to the “Mediterranean” dietary pattern showed a 40% increased likelihood of getting pregnant after IVF/ICSI treatment, also reflected by a high concentration of vitamin B6 and folate in their blood and follicular fluid. However, although the “health conscious–low processed” dietary pattern was positively related with the presence of folates in the red blood cells, it did not affect IVF/ICSI outcomes, suggesting that the “Mediterranean” diet seems to increase the chance of pregnancy following IVF/ICSI treatments.

These findings underline the importance of providing specific preconception counseling to women wanting to conceive, with reference to the development of nutritional interventions aimed at improving fertility treatment and success rates in assisted reproductive performance. However, more research and intervention studies are needed to explore the role of diet quality in ART protocols.

#### 2.1.2. Preconception Care Intervention Through Diet Normalization: In Vivo Animal Models

Preconception diet represents an important modifiable risk factor with the potential to be beneficial to fertility outcomes. Aligning dietary patterns with food-based dietary guidelines (FBDGs) allows one to reduce and limit the intake of free sugars and foods high in saturated fats, promoting instead the intake of base food products commonly high in components like vegetables, whole grains and fish rich in unsaturated fats [8,32].

Although studies involving dietary normalization in patients attempting natural conception are still limited, an evaluation of a preconception care intervention (PCCI) in animal models may provide a valuable contribution that can allow for a better understanding of this type of intervention.

In a study aimed to evaluate the effects of preconception diet, five-week-old female outbred Swiss mice were fed a control (CTRL) or high-fat (HF) diet for 7 weeks (7 w) [33]. After 7 weeks on an HF regimen, the mice were divided into four PCCI treatment subgroups, divided between those continuing the high-fat diet and switching to a normal control diet and subgroups that adopted a caloric-restricted control diet for 2, 4 or 6 weeks.

The results showed that the continuous HF diet led to oocytes with significantly a higher lipid droplet volume, higher reactive oxygen species (ROS) levels, mitochondrial dysfunction and increased mtDNA copy number, markers associated with reduced oocyte quality and fertility. After switching to a normal CTRL diet after 2 weeks, oocytes showed a lower lipid droplet content and increased mitochondrial activity, possibly correlated with enhanced lipid energy consumption and improved glucose tolerance in cumulus cells. At four and six weeks, mitochondrial efficiency further improved with a reduced ROS/active mitochondria ratio, suggesting enhanced metabolic efficiency and antioxidant capacity.

However, the substantial improvements were most notable after 6 weeks of the PCCI, indicating that the full cycle of folliculogenesis benefits from a prolonged non-high fat environment. Caloric restriction also reduced ROS levels over time, despite persistent lipid accumulation. Overall, the findings suggest that both the adopted strategies can mitigate HF-induced damage, but a 6-week intervention was the most effective in restoring oocyte quality, highlighting the pivotal influence of maternal diet prior to conception.

Furthermore, a PCCI was also evaluated in mares to assess metabolic changes and lipid profiles in ovarian follicular cells to determine the potential of dietary supplementation to attenuate these modifications. Twenty nonlactating light-horse mares were divided into normal-weight (NW), obese (OB) and obese supplemented-diet (OBD) groups and fed according to group-specific regimens for 6–10 weeks before follicular sample collection [34]. The OB mares showed a greater abundance of lipids in cumulus cells, elevated ROS production, mitochondrial damage and a metabolic shift from carbohydrate to fatty acid oxidation in granulosa cells, indicating a significant metabolic stress in the follicular environment due to obesity. Additionally, OB granulosa cells showed an increased expression of the Cytochrome P450 Family 19 Subfamily A Member 1 (*CYP19A1*) gene and higher levels of mitochondrial complexes I and III, crucial ROS production sites, possibly due to altered electron transport dynamics. In contrast, OBD granulosa cells showed normalized gene and protein expression, reduced ROS production, enhanced pyruvate oxidation and a reduced expression of antioxidant enzymes glutathione peroxidase 1 (*GPX1*) and superoxide dismutase 2 (*SOD2*), indicating that the supplementation may have exerted a stabilizing effect.

Altogether, these data could suggest that maternal obesity may induce lipotoxicity and metabolic stress in follicular cells, impairing oocyte quality. Targeted nutritional intervention may partially restore granulosa cell mitochondrial function and cumulus cell lipid balance, suggesting potential benefits for fertility.

Another study conducted on Western-type-diet-induced obese Swiss mice evaluated whether dietary normalization or a calorie-restricted diet for 2 or 4 weeks could improve metabolic health and oocyte quality [35]. Female mice were fed a control (CTRL) or a high-fat/high-sugar (HF/HS) diet for 7 weeks. Subsequently, the mice were assigned to a control diet (HF_CTRL) or to a 30% calorie-restricted diet (HF_CR) for 2 or 4 weeks. After 2 weeks of the PCCI, the cumulus cells of the HF_HF mice showed an upregulation of the insulin receptor substrate 1 (*Irs1*) and solute carrier family 2 member 1 (*Slc2a1*) genes, involved in the insulin signaling pathway and glucose metabolism, indicating metabolic stress. However, switching to the control diet normalized *Irs1* expression but not the expression of *Slc2a1*, which remained dysregulated. In contrast, HF_CR mice displayed a significantly higher expression of the Acyl-CoA dehydrogenase medium chain (*Acadm*), a gene involved in lipid metabolism, suggesting a shift in energy substrate use. The HF_CTRL mice showed an improved body weight, serum lipid profile and glucose tolerance, while insulin sensitivity remained impaired. The oocyte lipid content decreased, but mitochondrial defects persisted, indicating incomplete cell recovery. After 4 weeks, the HF_HF mice showed a downregulation of *Irs1* and *Slc2a1*, possibly due to prolonged metabolic stress. At the same time, cumulus cells from the HF_CTRL mice still showed an improved expression of *Slc2a1* and of catalase (*Cat*), an antioxidant gene. The HF_CTRL oocytes, in addition, showed improved mitochondrial activity and a reduction in mitochondrial ultrastructure abnormalities, despite an elevated lipid content. However, the HF_CR mice showed an increased expression of hyaluronan synthase 2 (*Has2*) and pentraxin 3 (*Ptx3*), related to cumulus expansion and oocyte quality, and of peroxiredoxin 6 (*Prdx6*), correlated with oxidative stress. In addition, the cholesterol concentration and insulin sensitivity of the HF_CR mice were totally restored after 2 weeks, but all their mitochondrial parameters remained impaired. However, the other metabolic parameters were restored only after 4 weeks of intervention.

These results support that HF/HS diets can impair oocyte and metabolic health, and PCCIs, particularly in terms of caloric restriction, can partially restore function, supporting their role as fertility interventions.

It is possible to speculate that PCCIs can mitigate the effects of obesity and HF/HS diets in animal models, with the possibility to improve metabolic health and oocyte quality. Nevertheless, due to the limitation of in vivo studies and the absence of univocal guidelines to formulate PCCIs, more investigations are necessary to improve our understanding of the effect of dietary interventions on female fertility, both in humans and animal models. Moreover, the need for further studies replicating the same experimental conditions on human samples is underlined throughout the scientific literature, in order to bypass the difficulties associated with data translation when transitioning from an animal to a human setting.

### 2.2. Diet and Modulation of Gene Expression: The Effect of n-3 Polyunsaturated Fatty Acids (PUFAs) on the Endometrium

Dietary patterns are involved in several complex nutritional interactions that impact overall health and well-being and represent a significant tool for understanding individual dietary behaviors [36]. Although the specific mechanisms by which certain food groups influence fertility are still unclear, inflammation is considered to play a key role. Inflammation is a physiological process triggered in response to an infection or injury. Nevertheless, sub-chronic inflammation can lead to adverse effects on fertility, interfering with cell signaling pathways, central for normal ovulatory function. It has also been associated with menstrual irregularities, endometriosis, implantation failure and recurrent miscarriage [16,37]. Dietary factors, including polyunsaturated fatty acids (PUFAs), polyphenols, plant-based proteins, fiber, vitamins and minerals, typical of the Mediterranean diet, appear to exert a positive influence on female fertility. However, the precise mechanisms linking diet to fertility have yet to be fully elucidated. Genomic, epigenomic and microbiological pathways may be involved [38,39,40].

#### Impact of *n*-3 Polyunsaturated Fatty Acid Supplementation on Endometrial Gene Expression

Polyunsaturated fatty acids, typical of the Mediterranean diet, seem to influence reproductive health through multiple mechanisms. PUFAs affect the biosynthetic pathways involved in both prostaglandin synthesis and steroidogenesis, both involved in the regulation of reproductive function. The PUFA composition of cell membranes reflects dietary intake and also plays a crucial role during fertilization [18,41].

Notably, *n*-3 PUFAs support membrane integrity, gene transcription and cell proliferation [42]. They are also precursors of the anti-inflammatory eicosanoids and of other key mediators, such as thromboxanes, prostacyclins, prostaglandins, resolvins, protectins, lipoxins, leukotrienes and maresins, which control not only the inflammatory response but also platelet aggregation and immunity [16].

Emerging evidence suggests that eicosapentaenoic acid (EPA) and docosahexaenoic acid (DHA) may suppress uterine prostaglandin F2A (PGF2A) synthesis, which regulates corpus luteum lifespan and potentially influences uterine function and embryo survival [43,44]. To evaluate the effect of the dietary inclusion of *n*-3 PUFAs on the uterine endometrial mRNA expression of key genes regulating PGF2A biosynthesis, beef heifers were fed with a low- or high-*n*-3-PUFA diet for 45 days [43]. After dietary supplementation, their endometrial tissues revealed increased EPA/DHA levels combined with a reduction in the arachidonic acid concentration. In addition, another series of markers including prostaglandin E synthase (*PTGES*), peroxisome proliferator activated receptor alpha (*PPARA*) and peroxisome proliferator activated receptor delta (*PPARD*) were upregulated, while phospholipase A2 group IIA (*PLA2G2A*) showed a trend toward downregulation. Overall, these results indicate that the expression of key genes involved in the biosynthesis of series-2 prostaglandins in the bovine uterus are regulated in response to dietary *n*-3 PUFA supplementation, potentially influencing uterine function and embryo survival. Moreover, *n*-3 PUFAs are ligands for PPAR A and D, possibly exerting a beneficial effect on fertility.

Under the same experimental conditions, *n*-3 PUFA supplementation in cattle altered endometrial gene expression related to prostaglandin synthesis, steroidogenesis, transcription factor regulation, immune modulation and tissue remodeling [45]. Particularly, the prostaglandin reductase 2 gene (*PTGR2*) was found to be downregulated following *n*-3 PUFA supplementation, while the endothelin 1 gene (*EDN1*) and the endothelin receptor type A (*EDNRA*), linked to PGF2A-mediated luteolysis, were both upregulated at the uterine level. The treated group also revealed an improved expression of the coiled-coil alpha-helical rod protein 1 (*CCHCR1*) gene, which presumably upregulates the transcription of the steroidogenic acute regulatory (*STAR*) gene. The estrogen receptor 1 (*ESR1*) and the insulin like growth factor 1 (*IGF1*) were found to be downregulated, while the scaffold attachment factor B (*SAFB*) gene was upregulated, suggesting a suppression of estrogen signaling during luteolysis.

Additionally, *n*-3 PUFA supplementation in cattle significantly modulated endometrial gene expression related to tissue development, as well as reproductive and immune function [46]. Among the most affected genes, the forkhead box D1 (*FOXD1*), forkhead box D3 (*FOXD3*), nuclear factor-kappa B1 (*NFKB1*), NK3 homeobox-1 (*NKX3-1*), forkhead box A2 (*FOXA2*), *PPARA* and secreted phosphoprotein 1 (*SPP1*) genes were recognized as potential regulators of gene expression in the endometrium of bovine supplemented with *n*-3 PUFA. Notably, *ESR1* and progesterone receptor (*PGR*) genes, two steroid hormones receptors crucial for reproductive function, were downregulated, potentially reducing the transcriptional activity of the *NFKB1* gene, a key player in inflammatory and immune responses. Additionally, the expression of the insulin like growth factor 2 (*IGF2*) gene increased, potentially positively affecting the establishment of pregnancy, while insulin-like growth factor 2 binding protein 3 (*IGF2BP3*) was reduced, possibly enhancing *IGF2*’s effects on the bovine endometrium. Following *n*-3 PUFA supplementation, *PPARA* and *PPARD* were upregulated, in contrast to peroxisome proliferator activated receptor gamma (*PPARG*), which remained unchanged, highlighting the *n*-3 PUFA’s influence on gene transcription and reproductive physiology.

In summary, these results demonstrated that *n*-3 PUFA supplementation in cattle diets considerably affects uterine endometrial gene expression, linking dietary intake to reproductive physiology and events. These effects appear to be mediated, at least in part, through the transcriptional regulation of multiple biological processes that may influence the uterine environment.

In breeding sows, *n*-3 PUFA supplementation through flaxseed oil altered endometrial gene expression during early pregnancy [47]. The results showed significant differences in the gene expression of prostaglandin F synthase (*PGFS*), *PTGES* and carbonyl reductase-1 (*CBR1*) genes. The expression level of *PTGES* transcripts during early pregnancy in the treated group was significantly higher over 10–11 days of pregnancy, suggesting that *n*-3 PUFA supplementation stimulates the synthesis of PGES enzymes during early porcine pregnancy. In contrast, the relative expression levels of *PGFS* and *CBR1* transcripts were significantly lower in the *n*-3 supplemented group, suggesting a consequent reduction in PGF2A production, which inhibits or delays the regression of the corpus luteum, and a modulation of the PGE2/PGF2A ratio, possibly favoring pre-implantation embryonic survival and better conditions for a successful pregnancy.

**Figure 2:**
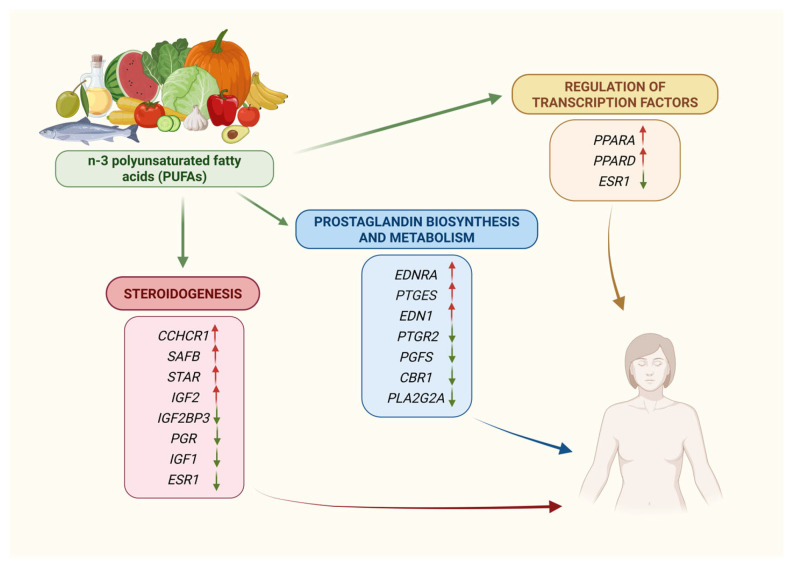
Impact of *n*-3 PUFA supplementation on endometrial gene expression. Red-coloured arrows (↑) stand for upregulation and green-coloured ones (↓) for downregulation.

Collectively, these data indicate that dietary *n*-3 PUFA supplementation can modulate the endometrial expression of the genes involved in the prostaglandin biosynthetic pathway during the first stages of gestation and, as a consequence, potentially influence a plethora of reproductive events, including implantation and embryonic survival (Figure 2).

The mechanisms by which dietary *n*-3 PUFA affects gene expression are complex and incorporate several processes. However, *n*-3 PUFA supplementation in diets could provide new resources for the development of nutritional management strategies to improve reproductive efficiency.

However, based on the literature mentioned, further large-scale studies are required to validate these effects and to include detailed functional analyses that would support their physiological relevance.

### 2.3. Physical Activity

**Figure 3:**
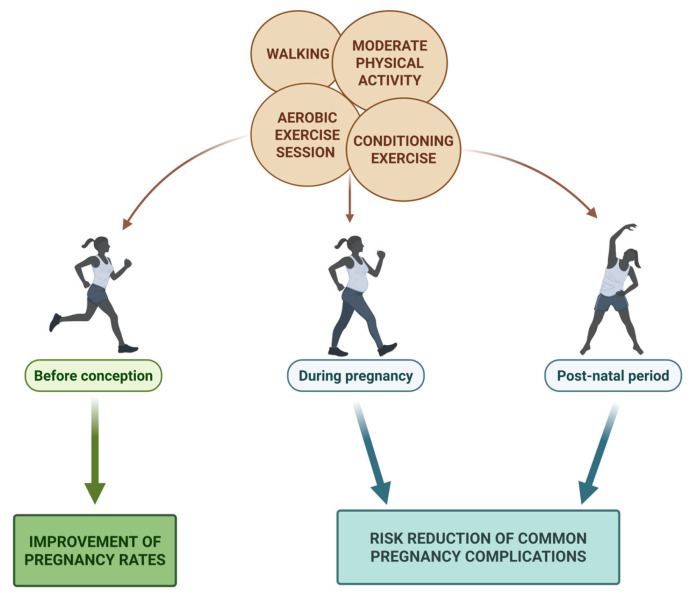
Pre- and post-conception impact of physical activity on female reproductive health.

According to the World Health Organization (WHO) [48], PA is defined as any movement produced by the skeletal muscles that requires energy expenditure, encompassing both daily activities and leisure movement. PA is beneficial to health and wellness, reducing the risk for noncommunicable diseases and poor health outcomes that result in a burden on the healthcare system. Regular physical exercise has the potential to improve cardiovascular health, manage weight and its associated comorbidities and can improve longevity [49]. Increased PA and decreased sedentary behavior may enhance fertility in women through the maintenance of body weight and hormone levels, which are indispensable for an improved probability of conception [50,51]. Conversely, high levels of PA may inhibit ovulation, resulting in decreased fertility [52,53,54]. It has been suggested that a combination of walking, moderate physical activity, aerobic exercises and conditioning exercises carried out for at least 30 min a day for a minimum of 150–200 min per week represents an important tool that may improve systemic and reproductive health [49,55,56,57,58]. These levels of exercise are recommended before pregnancy, during gestation and over the postpartum period. They have been shown to help reduce the risks of common complications of pregnancy and the postnatal period, such as gestational weight gain, gestational diabetes and postpartum depression [49,59,60,61,62]. However, these guidelines do not provide further information on how the type, intensity or duration of PA may alter fertility status [53]. In accordance with the WHO [48], it is strongly recommended that women who are physically active or habitually engaged in vigorous-intensity aerobic activity prior to pregnancy should continue this activity during gestation and the postpartum period. The association of exercise and dietary-related modifications, moreover, has shown an important improvement of pregnancy rates in women with reproductive health problems, indicating that PA intervention may be as effective as other generally clinical intervention strategies used to improve reproductive health outcomes [63]. However, an improvement in the outcomes may be not necessarily associated with weight loss, suggesting a beneficial effect of PA even in the absence of significant weight management [64,65] (Figure 3).

It has also been reported that PA can influence assisted reproduction. A longitudinal study involving 107 infertile women undergoing IVF treatment investigated the association between PA and sedentary behavior before and during treatment for 14 consecutive days. The results showed a positive correlation between PA and a lower amount of time spent sedentary before starting ART, obtaining a higher number of oocytes and embryos per controlled ovarian stimulation (COS). However, activity levels before and during IVF did not affect implantation, pregnancy or live birth rates, suggesting that PA does not affect the quality of the oocytes, but the quantity of those obtained [66].

Furthermore, a meta-analysis conducted on 3683 infertile couples undergoing IVF/ICSI treatments revealed that maternal PA before the treatment was associated with better assisted reproductive outcomes, mostly based on increased rates of clinical pregnancies and live births [67]. Nevertheless, a minor but not statistically significant increase in the implantation rate was observed, whereas the miscarriage rate was not associated with PA in the women before the ART cycles. These results suggested that PA before IVF/ICSI treatments may help women improve their reproductive outcomes and enhance their chance of a successful pregnancy.

In support, Mena et al. [64] grouped eighteen studies in a systematic review and meta-analysis, revealing that PA may improve pregnancy rates in women with reproductive health problems. In addition, PA interventions may be as efficient as other typical clinical intervention strategies for improving reproductive health outcomes.

The mechanisms whereby PA in previous IVF/ICSI cycles improve pregnancy outcomes could be complex, and no molecular pathways have yet been identified. Regular PA seems to be related to an effect on the clinical pregnancy rate, which may be due to an improvement in health status through changes in energy balance [68] and possibly an improvement through insulin sensitization, the restoration of ovarian function and the sensitization of the ovary to clomiphene citrate during simple ovulation induction [69,70]. Furthermore, regular PA can help alleviate stress and anxiety, which have been shown to be a significant risk factor affecting assisted reproductive outcomes [71].

The type, intensity, frequency and duration of optimal PA interventions in order to benefit reproductive potential remain unclear. However, it has been suggested that physical training may represent an affordable and feasible alternative or complementary therapy to fertility treatments for both partners to ameliorate fertility outcomes [72,73].

Nevertheless, the current literature remains limited to comprehensively elucidate the influence of PA on the molecular signature of the female reproductive system prior to conception. Further research is needed to elucidate the pathways involved in these mechanisms and their potential implications for reproductive health and fertility outcomes.

## 3. Conclusions

Infertility is a multifactorial condition affecting a growing number of individuals worldwide and can be significantly influenced by modifiable lifestyle factors such as nutrition, physical activity and body composition.

The increased body of knowledge linking this type of intervention to an enhancement of fertility underlines the importance of developing new preventive multidisciplinary approaches, especially at a preconception stage.

Evidence suggests that adherence to dietary patterns like the “Mediterranean” and “pro-fertility” diets may increase the chances of getting pregnant and having a live birth, likely due to anti-inflammatory and antioxidant mechanisms, weight regulation and hormonal balance. On the contrary, Western-style diets, often rich in saturated fats, sugar and refined grains, may provoke systemic inflammation and metabolic dysfunction, negatively impacting fertility potential.

A series of dietary factors, known as *n*-3 polyunsaturated fatty acids, typical supplements of the Mediterranean diet, are gaining increasing attention for their potential benefits on reproductive outcomes. They have been found to be primarily involved in biosynthetic pathways, including prostaglandin synthesis and steroidogenesis, as well as mitigating metabolic stress and inflammation in reproductive tissues and cells.

*n*-3 PUFAs can also interact with transcription factors like PPARs, which translate nutritional stimuli into gene expression modifications and, for this reason, could represent new nutraceutical alternatives for the development of strategies to improve reproductive efficiency.

Nevertheless, although the current literature on animal studies and limited human data highlight a positive influence on oocyte quality and endometrial gene regulation, further detailed research is necessary to deeply describe functional outcomes and clinical fertility improvements.

In parallel, physical activity represents another pivotal tool for reproductive health. Moderate PA has shown potential to support hormonal regulation, reduce stress and insulin resistance and ultimately improve ovarian function and, consequently, pregnancy and live birth rates. When combined with dietary modifications, physical activity may further enhance the effects of lifestyle interventions.

Preconception counseling that integrates nutritional education and weight management strategies offers a valuable opportunity to support women in achieving optimal reproductive health. Moreover, a personalized approach that includes lifestyle modifications may not only increase the possibility of conception but also promote a healthier pregnancy journey.

A deeper comprehension of the interlink between nutrition, lifestyle habits and fertility could contribute to the development of evidence-based guidelines for infertile patients, paving the way forward beyond general health recommendations. Moreover, preconception programs aimed at promoting positive lifestyle and nutritional choices should be considered as first-line treatments for unexplained infertility.

However, the limited data about in vivo studies and a lack of standardized guidelines represent challenges that must be overcome.

In light of the rising fertility issues among women, coordinated lifestyle interventions should be considered a core component of reproductive care, offering measurable benefits for both fertility and overall well-being.
